# Assembly of tetraspanins, galectin-3, and distinct N-glycans defines the solubilization signature of seminal prostasomes from normozoospermic and oligozoospermic men

**DOI:** 10.48101/ujms.v126.7673

**Published:** 2021-09-03

**Authors:** Tamara Janković, Jelena Danilović Luković, Irena Miler, Ninoslav Mitić, Ljiljana Hajduković, Miroslava Janković

**Affiliations:** a University of Belgrade, Institute for the Application of Nuclear Energy, INEP, Belgrade, Serbia; b University of Belgrade, Institute of Nuclear Sciences, VINČA, National Institute of the Republic of Serbia, Belgrade, Serbia

**Keywords:** Extracellular vesicles, detergent sensitivity, CD63, CD9, gamma-glutamyl transferase, molecular patterns, normozoospermia, oligozoospermia

## Abstract

**Background:**

Prostasomes, extracellular vesicles (EVs) abundantly present in seminal plasma, express distinct tetraspanins (TS) and galectin-3 (gal-3), which are supposed to shape their surface by an assembly of different molecular complexes. In this study, detergent-sensitivity patterns of membrane-associated prostasomal proteins were determined aiming at the solubilization signature as an intrinsic multimolecular marker and a new parameter suitable as a reference for the comparison of EVs populations in health and disease.

**Methods:**

Prostasomes were disrupted by Triton X-100 and analyzed by gel filtration under conditions that maintained complete solubilization. Redistribution of TS (CD63, CD9, and CD81), gal-3, gamma-glutamyltransferase (GGT), and distinct N-glycans was monitored using solid-phase lectin-binding assays, transmission electron microscopy, electrophoresis, and lectin blot.

**Results:**

Comparative data on prostasomes under normal physiology and conditions of low sperm count revealed similarity regarding the redistribution of distinct N-glycans and GGT, all presumed to be mainly part of the vesicle coat. In contrast to this, a greater difference was found in the redistribution of integral membrane proteins, exemplified by TS and gal-3. Accordingly, they were grouped into two molecular patterns mainly consisting of overlapped CD9/gal-3/wheat germ agglutinin-reactive glycoproteins and CD63/GGT/concanavalin A-reactive glycoproteins.

**Conclusions:**

Solubilization signature can be considered as an all-inclusive distinction factor regarding the surface properties of a particular vesicle since it reflects the status of the parent cell and the extracellular environment, both of which contribute to the composition of spatial membrane arrangements.

## Introduction

Tetraspanin-web and galectin-glycoprotein lattices represent distinct multi/macromolecular complexes assembled at the plasma membrane and are supposed to facilitate different biological activities/functions (1–3). Extracellular vesicles (EVs), membranous structures originating from plasma- or intracellular membranes, are considered enriched in tetraspanins (TS), which are used as canonical markers (4). Regarding the presence of lectins, including galectins, although not widely studied in this context, there are data indicating that galectin-3 (gal-3) is involved in the biogenesis of EVs and can be used as a reliable marker (5). Both TS and gal-3 are thought to shape not only the surface of EVs but also the cargo composition (6).

Prostasomes, EVs originating from the prostate and abundantly present in human seminal plasma (SP), are reported to express TS: CD63, CD9, and CD81, as well as gal-3 (2, 7–9). In addition, gal-3 and mannosylated/sialylated glycans were found to contribute to the prostasomal surface in a specific way in terms of accessibility and native presentation which could be altered in pathological conditions associated with male fertility (2). This study aimed to delineate the positions of selected TS: CD63, CD9, and CD81, that is, to obtain data on their molecular associations and relate them to gal-3 and selected N-glycans, all known to reside on the surface of prostasomes. Although of possible general importance for the identification of distinct vesicle types and their functions in different heterogeneous extracellular landscapes, related data are still missing. Thus, it is presumed that solubilization signature is an all-inclusive distinction factor regarding surface properties of a particular vesicle, since it can reflect the status of the parent cell and the extracellular environment, both of which contribute to the composition of spatial membrane arrangements. By establishing solubilization signature, we aimed at determining a new qualitative data suitable as a reference for the comparison of any type of vesicles.

The existence of distinct prostasomal surface molecular complexes was deduced from their detergent resistance (revealing TS-primary ligand association, gal-3-glycoprotein association, and insoluble membranes) or detergent sensitivity (revealing solubilized glycoprotein–glycolipid complexes). Related molecular patterns established from the mode of response to disruption by a non-ionic detergent of high stringency were used as a reference to annotate and/or compare prostasomal preparations from normozoospermic and oligozoospermic men.

In general, this approach is readily applicable, and it is not supposed to be significantly affected by different isolation procedures. Getting insight into the distribution patterns of TS on different membrane domains can add new value to their common use (based on presence only) as EVs markers. Moreover, since TS have distinct biological activities involved in cell adhesion, motility, and metastasis, as well as cell activation and signal transduction (10, 11), possible differences in their organization may have biomedical consequences. Thus, solubilization signatures of EVs might relate their structure with functional alterations in distinct pathological conditions.

## Material and methods

### Materials

Monoclonal anti-CD63 antibody (clone TS63) was from Abcam (Cambridge, UK), monoclonal anti-CD81 (clone M38) and monoclonal anti-CD9 (clone MEM-61) were from Invitrogen by Thermo Fisher Scientific (Carlsbad, CA, USA), and biotinylated goat anti-galectin-3 (gal-3) antibodies were from R&D Systems (Minneapolis, USA). 3,3′,5,5′-tetramethylbenzidine (TMB), bovine serum albumin (BSA), and Triton X-100 (TX-100) were from Sigma (St. Louis, MO, USA). Biotinylated goat anti-mouse IgG, biotinylated plant lectins: Con A (Concanavalin A), wheat germ agglutinin (WGA), and the Elite Vectastain ABC kit were from Vector Laboratories (Burlingame, CA, USA). Sephadex G-200 was from Pharmacia AB (Uppsala, Sweden). The silver stain kit and SDS-PAGE molecular mass standards (broad range) were from Bio-Rad (Hercules, CA, USA). Nitrocellulose membrane and Pierce ECL Western Blotting Substrate were from Thermo Scientific (Rockford, IL, USA). Microwell plates were from Thermo Scientific (Roskilde, Denmark).

### Human semen samples

This study was performed on the leftover, anonymized specimens of human semen taken for routine analysis, and since existing human specimens were used, it is not considered as research on human subjects. It was approved by the institutional ethics committee according to the guidelines (No. # 02-832/1), which conforms to the Helsinki Declaration, 1975 (revised 2008). Sperm parameters were assessed according to the recommended criteria of the World Health Organization (released in 2010.), concerning numbers, morphology, and motility.

Sperm cells and other debris were removed from the ejaculate by centrifugation at 1,000 × g for 20 min.

### Isolation of prostasomes from human seminal plasma

Two pools of human SP of normozoospermic men and two pools of human SP of oligozoospermic men were used for the isolation of prostasomes. Each pool contained 10 individual SP samples. Prostasomes from normozoospermic men (sPro-N) and oligozoospermic men (sPro-O) were isolated from SP according to the modified protocol of Carlsson et al. (12). CD63-, CD9-, and CD81-immunoreactivities were used as the indicator of EVs’ presence. These prostasomal preparations were subjected to detergent treatment by incubation with 1% TX-100 for 1 h at room temperature and then subjected to gel filtration as the method of choice (13, 14). We monitored the redistribution of selected markers during gel filtration as an indicator of release from vesicles using the combined analysis of intact fractions (solid-phase assay with immobilized fractions and microscopy) and methods analyzing denatured fractions (electrophoresis and lectin blot).

### Gel filtration

Gel filtration separation profiles of TX-100-treated prostasomes were obtained under conditions where TX-100 was present during elution to ensure maintenance of total solubilization (15). Thus, the detergent-treated seminal prostasome preparation (1 mL) was loaded on a Sephadex G-200 column (bed volume 35 mL) equilibrated and eluted with 0.03 M Tris-HCl, pH 7.6, containing 0.13 M NaCl and 1% TX-100. Fractions of 1 mL were collected. The elution was monitored as described previously (16). Briefly, gel filtration-separated fractions were coated on microwell plates at 4°C overnight. After washing steps (3 × 300 μL with 0.05 M phosphate-buffered saline, PBS), they were blocked with 50 μL 1% BSA for 1.5 h and then washed again. Biotinylated plant lectins: Con A and WGA (50 μL, 0.5 mg/mL) were allowed to react for 30 min at room temperature, washed out, and followed by the addition of 50 μL of avidin/biotin–HRPO complex (Elite, Vectastain ABC Kit, prepared according to the manufacturer’s instructions). After incubation for 30 min, at room temperature, the plates were rinsed and developed using 50 μL TMB substrate solution. The reaction was stopped with 50 μL 2 N sulfuric acid. Absorbance was read at 450 nm using a Wallac 1420 Multilabel counter Victor3V (Perkin Elmer, Waltham, MA, USA). The elution profile of gamma-glutamyl transferase (GGT) was monitored by measuring enzyme activity using GGT colorimetric assay kits (Bioanalytica, Madrid, Spain), according to the manufacturer’s instructions for Biosystems A25 (Barcelona, Spain). The selected fractions were further analyzed by electrophoresis and blotting.

Native seminal prostasome preparations were analyzed in the same way except that TX-100 was not added to the elution buffer.

### SDS-PAGE

Corresponding samples were resolved on 10% separating gel with 4% stacking gel under denaturing and reducing conditions (17) and stained with silver nitrate, using a silver stain kit (Bio-Rad) according to the manufacturer’s instructions. The gel was calibrated with SDS-PAGE molecular weight standards (broad range).

### Western blot and dot blot

Samples were transferred onto nitrocellulose membrane by semi-dry blotting using a Trans-blot SD (Bio-Rad Laboratories). The conditions were as follows: transfer buffer, 0.025 M Tris containing 0.192 M glycine and 20% methanol, pH 8.3 under a constant current of 1.2 mA/cm^2^ for 1 h. The membrane was blocked with 3% BSA in 0.05 M PBS, pH 7.2, for 2 h at room temperature, and then used for lectin-blotting (1) as described below.

For dot blot, 3 μL of each corresponding fraction was applied to the nitrocellulose membrane, dried, blocked as described above, and subjected to immunoblotting (2).

1. Lectin blotting
  Lectin blotting was performed as described earlier (18). The membrane was incubated with the chosen biotinylated plant lectin (0.2 μg/mL in 0.05 M PBS, pH 7.2) for 1 h at room temperature and then washed six times in 0.05 M PBS, pH 7.2. Avidin/biotinylated horseradish peroxidase (HRPO) from Vectastain Elite ABC kit (prepared according to the manufacturer’s instructions) was added and incubated for 30 min at room temperature. The membrane was then rinsed again six times in 0.05 M PBS, pH 7.2, and the proteins were visualized using Pierce ECL substrate solution (Thermo Scientific, Rockford, IL, USA), according to the manufacturer’s instructions.
2. Immunoblotting
  Immunodot blot was performed as previously established (18). For immunoblotting, the membrane was incubated with the corresponding antibodies: anti-CD63 antibody (0.5 μg/mL), anti-CD81 antibody (0.25 μg/mL), anti-CD9 antibody (0.5 μg/mL), and biotinylated anti-gal-3 antibodies (0.025 μg/mL), overnight at 4°C. After a washing step, bound antibody was detected by incubation with biotinylated goat anti-mouse IgG for 30 min at room temperature. The membrane was rinsed and the avidin/biotinylated HRPO mixture from the Elite Vectastain ABC kit was added, followed by incubation for 30 min at room temperature. After another washing step, the blots were visualized using Pierce ECL Western blotting substrate according to the manufacturer’s instructions.

### Transmission electron microscopy (TEM)

TEM was performed as described previously (19). Samples were applied to the formvar-coated, 200 mesh, Cu grids by grid flotation on 10 μL sample droplets, for 45 min at room temperature. This was followed by steps of fixation (2% paraformaldehyde, 10 min), washing (PBS, 3 ***×*** 2 min), post-fixing (2% glutaraldehyde, 5 min), and a final wash with distilled H_2_O (2 min). Grids were then air-dried, and the images were collected using a Philips CM12 electron microscope (Philips/FEI, Eindhoven, the Netherlands).

## Results

### Prostasomes from human seminal plasma of normozoospermic men: influence of TX-100 treatment

**Figure 1:**
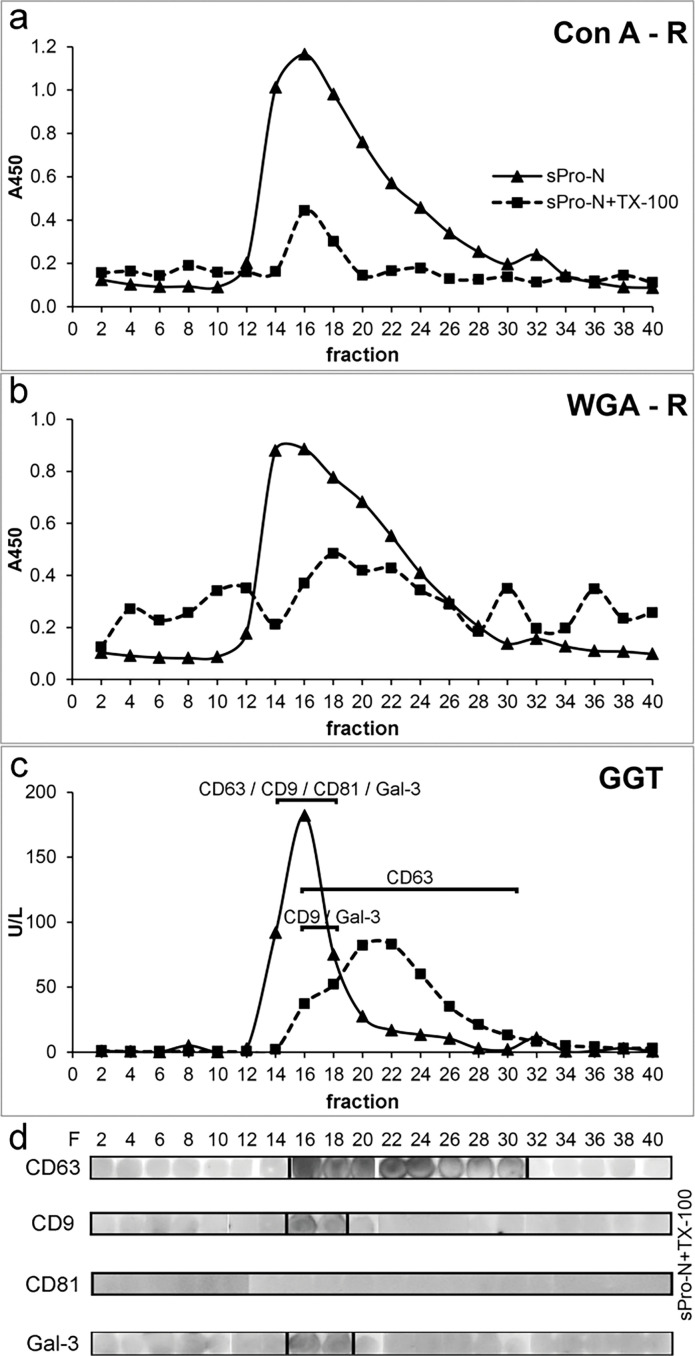
Surface-associated glycoproteins and gamma-glutamyl transferase on the seminal prostasomes of normozoospermic men: influence of detergent treatment. Prostasomes from human seminal plasma of normozoospermic men (sPro-N) were subjected to Triton X-100 (TX-100) treatment followed by gel filtration on a Sephadex G-200 column. Reference elution profiles of native sPro-N (eluted at void volume) were shown for comparison. Elution of (a) concanavalin A-reactive glycans (Con A-R) and (b) wheat germ agglutinin-reactive glycans (WGA-R). (c) Elution of GGT. (d) Distribution of tetraspanins (CD63, CD9, and CD81) and gal-3 (indicated in panel c) was monitored by measuring the immunoreactivity of dot blot-immobilized fractions. The presence of TX-100 in eluted fractions caused spilled appearance of dots and background staining. A450: absorbance at 450 nm; GGT: gamma-glutamyl transferase activity expressed in U/L, unit per liter; gal-3: galectin 3; F: fraction.

Distributions of distinct surface-associated markers of prostasomes from human SP of normozoospermic men (sPro-N) after solubilization with TX-100 are shown in Figure 1a–d. In general, their gel filtration elution positions differed in terms of a more or less noticeable shift from the void volume where they were co-eluted on native vesicles, revealing new patterns of associations.

**Figure 2:**
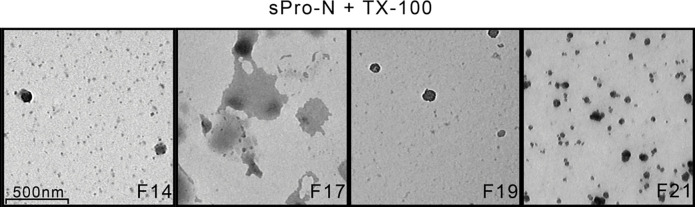
Transmission electron microscopy of the detergent-treated prostasomes from human seminal plasma of normozoospermic men. Ultrastructural appearance of prostasomes from human seminal plasma of normozoospermic men (sPro-N) treated with TX-100. Selected gel filtration-resolved fraction (F) was shown (Figure 1). Bar 500 nm. F14: rare vesicles; F17: broken vesicles surrounded with leaking content; F19: vesicles with disrupted irregular surface with no associated material; F21: irregular deposits.

**Figure 3:**
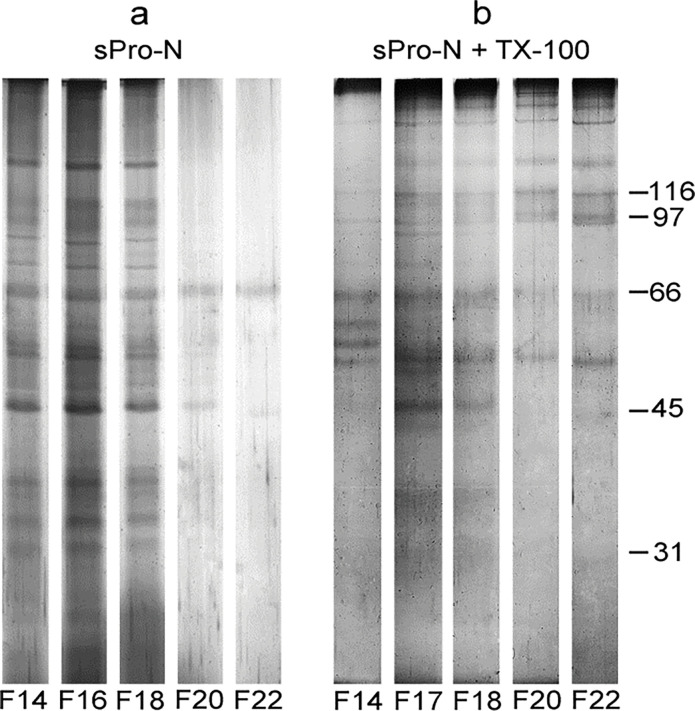
Protein composition of the detergent-treated prostasomes from human seminal plasma of normozoospermic men. Selected gel filtration (Figure 1) fractions (F) of native prostasomes from human seminal plasma of normozoospermic men (sPro-N) (a) and TX-100 treated sPro-N (b) were resolved by electrophoresis and stained with silver. Although aggregation is present as judged by intense bands at the border of the stacking and separating gel, the referent protein bands are preserved. No adjustment of glycoprotein content (equal concentration per lane) was made, that is, the eluted fractions were loaded as such (equal volume) to keep on elution profiles. The numbers (in kDa) indicate the position of molecular mass standards.

**Figure 4:**
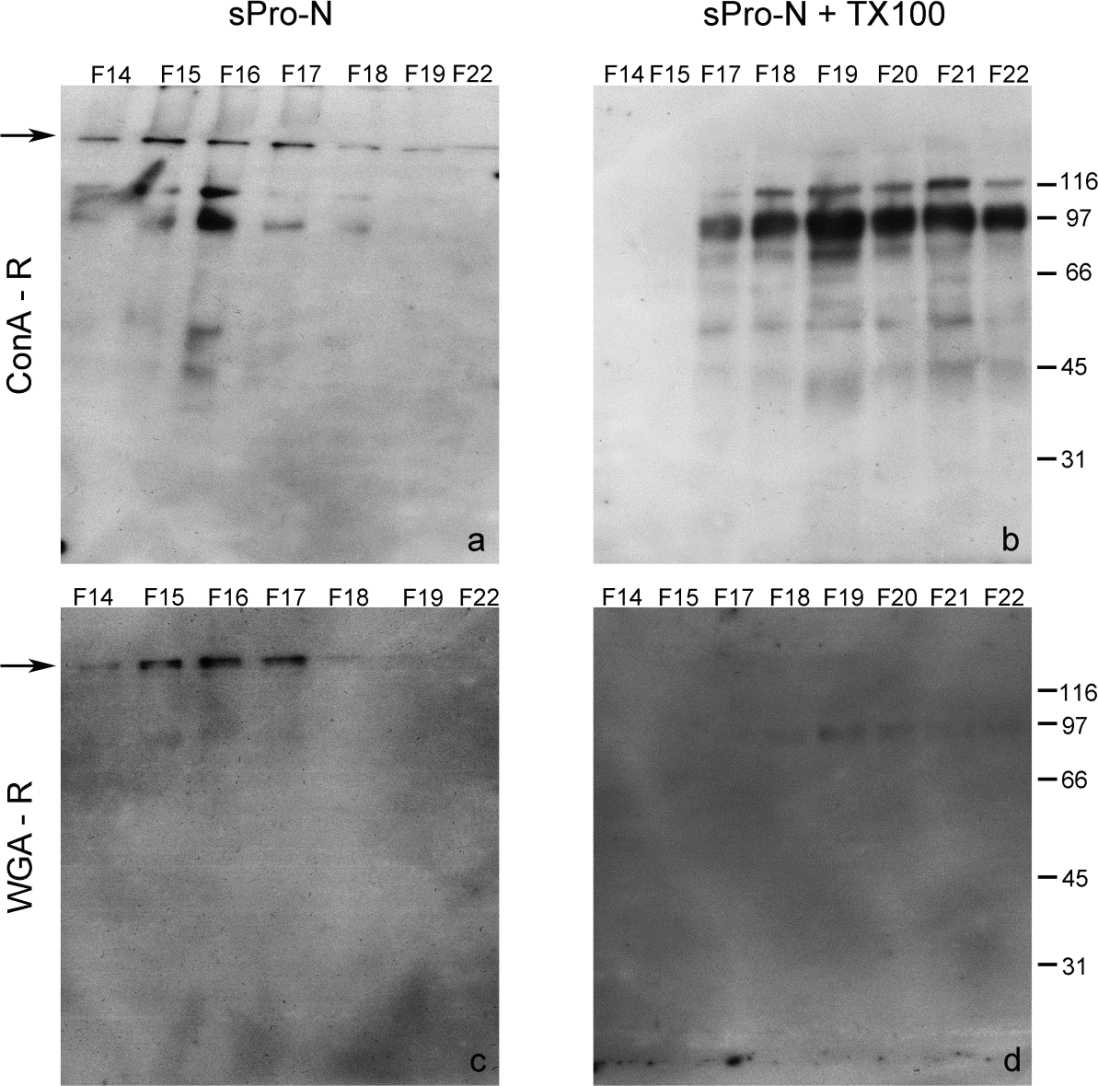
Distribution of Con A-reactive and WGA-reactive glycoproteins of the detergent-treated seminal prostasomes from normozoospermic men. Selected gel filtration (Figure 1) fractions (F) of native prostasomes from human seminal plasma of normozoospermic men (sPro-N) (a, c) and TX-100 treated sPro-N (b, d) were subjected to lectin-blot using Con A (a, b) and WGA (c, d). Con A-R: concanavalin A-reactive glycoproteins; WGA-R: wheat germ agglutinin-reactive glycoproteins. The numbers (in kDa) indicate the position of molecular mass standards. Arrows indicate the border of stacking and separating gel.

In eluted fractions, total mannosylated and sialylated glycans, which can reside on both glycoprotein and glycolipids, were monitored by lectin-binding reactivity (Figure 1a, b), and the GGT as an individual protein marker was monitored by enzymatic activity (Figure 1c). Thus, Con A-reactive glycoproteins were solubilized as evidenced by a striking decrease at the initial position (Figure 1a). However, the related redistribution can be rather deduced than clearly shown according to the reactivity of fractions in the included column volume, possibly due to the influence of their structure on immobilization. In contrast to this, WGA-reactive glycoproteins seemed to be partly solubilized and released as aggregated, judging by the corresponding elution profile revealing broad peaks entering the column and trailing down along the entire chromatogram (Figure 1b). Moreover, they produced a small but distinct peak before that of intact vesicles, which suggests formation of larger protein complexes. As for GGT, it was also clearly solubilized from vesicles with TX-100 and exhibited an elution profile distinct from the examined glycans (Figure 1c). The influence of TX-100 on the distribution of TS: CD63, CD9, and CD81, chosen as integral membrane proteins, and gal-3, chosen as a soluble but membrane-associated molecule, was monitored by the immunodot blot as adequate method (in contrast to western blot) for monitoring surface-associated changes (Figure 1d). TS and gal-3 co-localized on native vesicles at a position overlapping the detected Con A- and WGA-reactive glycoproteins and GGT (Figure 1c, data not shown). After TX-100 treatment, CD63 was clearly released and exhibited broad distribution (Figure 1d, fractions 16–30), but also remained close to its initial position. In contrast to this, CD9 (Figure 1d, fractions 16–18) and gal-3 (Figure 1d, fractions 16–18) retained narrow distributions, that is, they were slightly shifted during elution. Moreover, their patterns overlapped completely. As for CD81, it could not be detected after detergent treatment (Figure 1d). To further follow up the influence of TX-100 on prostasomes, changes in their ultrastructure were analyzed (Figure 2). In the region where all examined markers remained more or less co-localized after TX-100 treatment, the microscopic inspection revealed the presence of structures that correspond to reorganized detergent-resistant domains of vesicular membranes. They appeared as broken vesicles surrounded with leaking content or smaller vesicles with disrupted irregular surfaces seemingly shrunken with no associated material. In contrast to this, in the region where released glycoproteins were separated (Figure 1, fractions 20–22), no such structures were visible, only irregular, possibly, protein deposits. To complement the results obtained for detergent-treated samples as such, changes in the patterns of total prostasomal glycoproteins were analyzed under denaturing and reducing conditions by electrophoresis (Figure 3) and lectin blot (Figure 4). Compared to the native vesicles (Figure 3a), in the TX-100-treated ones (Figure 3b), the proteins exhibiting a prostasome-like pattern remained clustered close to the initial position, that is, they were marginally shifted in elution. However, their abundance was noticeably lower. Specifically, discrete changes in terms of the abundance of major bands in the region below 66 kDa (encompass masses of TS and gal-3) were detected. In addition, clear loss of bands in the region corresponding to the masses of prostasomal signature bands (90–150 kDa) was also detected. Consequently, some of them were visible as shifted in a cluster of protein bands included in column volume (Figure 3b, fractions 20–22).

In contrast to proteins, prostasomal glycoproteins were considerably reorganized after TX-100 treatment. Regarding Con A-reactive glycoproteins, what the lectin-binding assay suggested was confirmed by the lectin blot (Figure 4). Thus, the pattern of Con A-reactive glycoproteins from native vesicles comprised high molecular mass band at the border of the stacking and separating gel and smear band in the stacking gel as well as four distinct lower molecular mass bands (Figure 4 a, fractions 15–17). After TX-100 treatment, a striking loss of high molecular mass components was observed. It can be related to different modes of redistribution of distinct lower molecular mass Con A-reactive bands. Specifically, the major 97 kDa band remained partly close to the initial position (Figure 4b, fraction 17) but was also released, that is, redistributed (Figure 4b, fractions 19–22). In addition, almost complete release of those with molecular masses below 66 kDa was observed (Figure 4a, b, fractions 15–17).

The pattern of WGA-reactive glycoproteins of native vesicles was comparable with that of the Con A-reactive ones in high molecular mass region (Figure 4c). However, after TX-100 treatment, their patterns were strikingly different (Figure 4d), as initially observed in the corresponding lectin-binding assay (Figure 1b). Thus, the high molecular mass band was completely lost, and a shift of weak 97 kDa was also detected.

Taken together, the profiles related to the cluster of Con A- and WGA-binding glycoproteins might be influenced by their presence in different but overlapping complexes. Moreover, it is possible that diverse, but overlapped, bands with matching molecular mass and lectin binding were detected, as indicated by disparate/selective presence/abundance of particular ones in the subsequently eluted fraction.

### Prostasomes from human seminal plasma of oligozoospermic men: influence of TX-100 treatment

**Figure 5:**
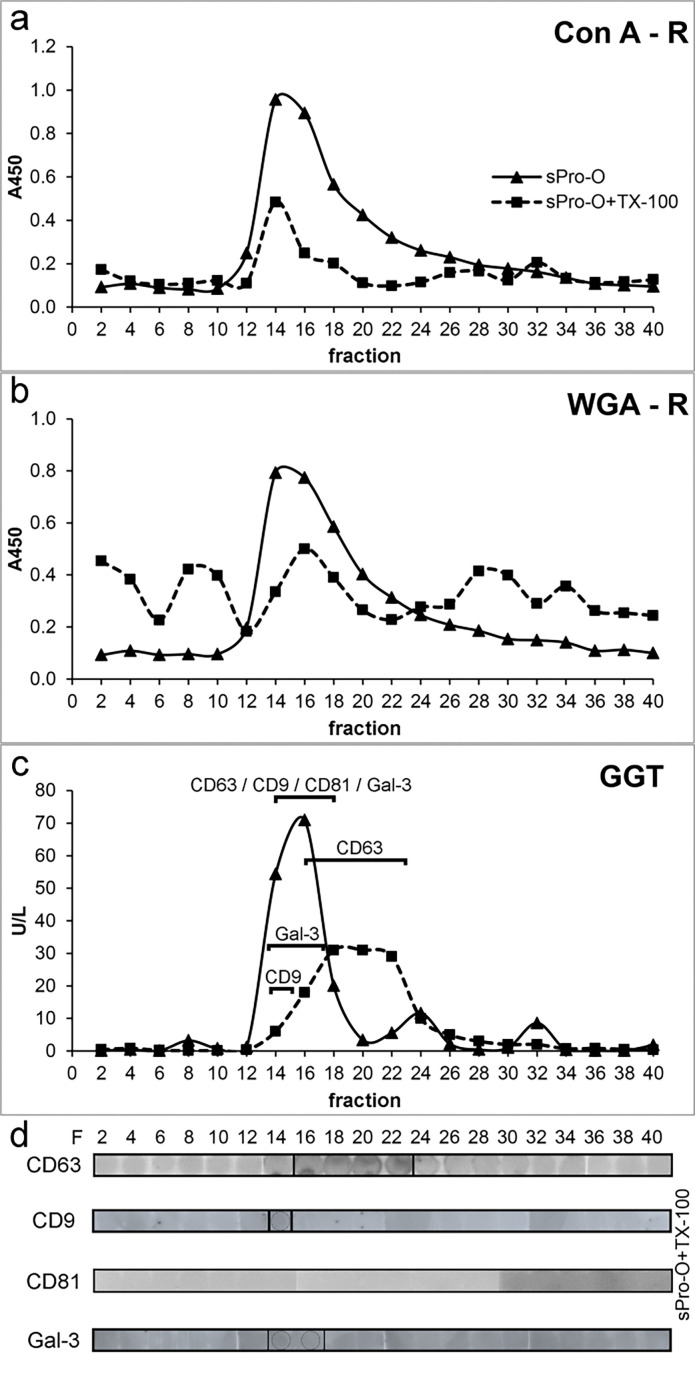
Surface-associated glycoproteins and gamma-glutamyl transferase on seminal prostasomes of oligozoospermic men: influence of detergent treatment. Prostasomes from human seminal plasma of oligozoospermic men (sPro-O) were subjected to Triton X-100 (TX-100) treatment followed by gel filtration on a Sephadex G-200 column. Reference elution profiles of native sPro-O from Sephadex G-200 column (eluted at void volume) were shown for comparison. Elution of (a) concanavalin A-reactive glycans (Con A-R) and (b) wheat germ agglutinin-reactive glycans (WGA-R). (c) Elution of GGT. (d) Distribution of tetraspanins (CD63, CD9, and CD81) and gal-3 (indicated in panel c) was monitored by measuring the immunoreactivity of dot blot-immobilized fractions. The presence of TX-100 in eluted fractions caused spilled appearance of dots and background staining. Barely detectable CD9- and gal-3-immunoreactivity is indicated by circle. A450, absorbance at 450 nm; GGT, gamma-glutamyl transferase activity expressed in U/L, unit per liter; gal-3, galectin 3; F, fraction.

**Figure 6:**
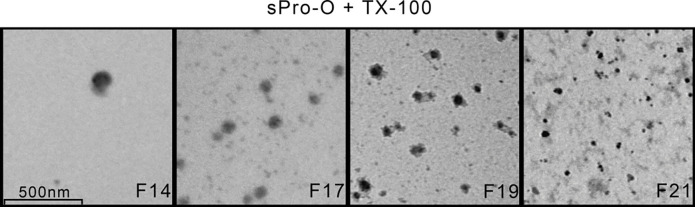
Transmission electron microscopy of the detergent-treated prostasomes from human seminal plasma of oligozoospermic men. Ultrastructural appearance of prostasomes from human seminal plasma of oligozoospermic men (sPro-O) treated with TX-100. Selected gel filtration-resolved fraction (F) was shown (Figure 5). Bar 500 nm. F14–F17: rare vesicles; F19–F21: irregular deposits.

**Figure 7:**
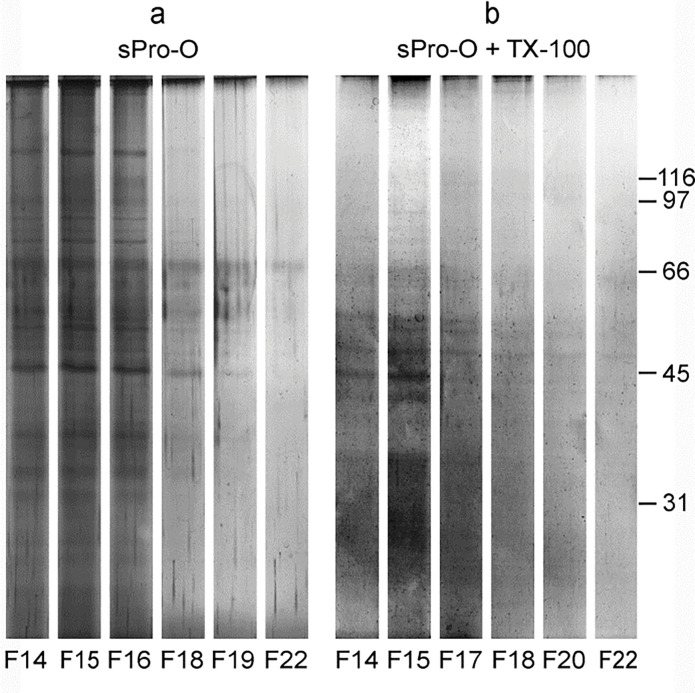
Protein composition of the detergent-treated prostasomes from human seminal plasma of oligozoospermic men. Selected gel filtration (Figure 5) fractions (F) of native prostasomes from human seminal plasma of oligozoospermic men (sPro-O) (a) and TX-100-treated sPro-O (b) were resolved by electrophoresis and stained with silver. No adjustment of glycoprotein content (equal concentration per lane) was made, that is, the eluted fractions were loaded as such (equal volume) to keep on elution profiles. The numbers (in kDa) indicate the position of molecular mass standards.

**Figure 8:**
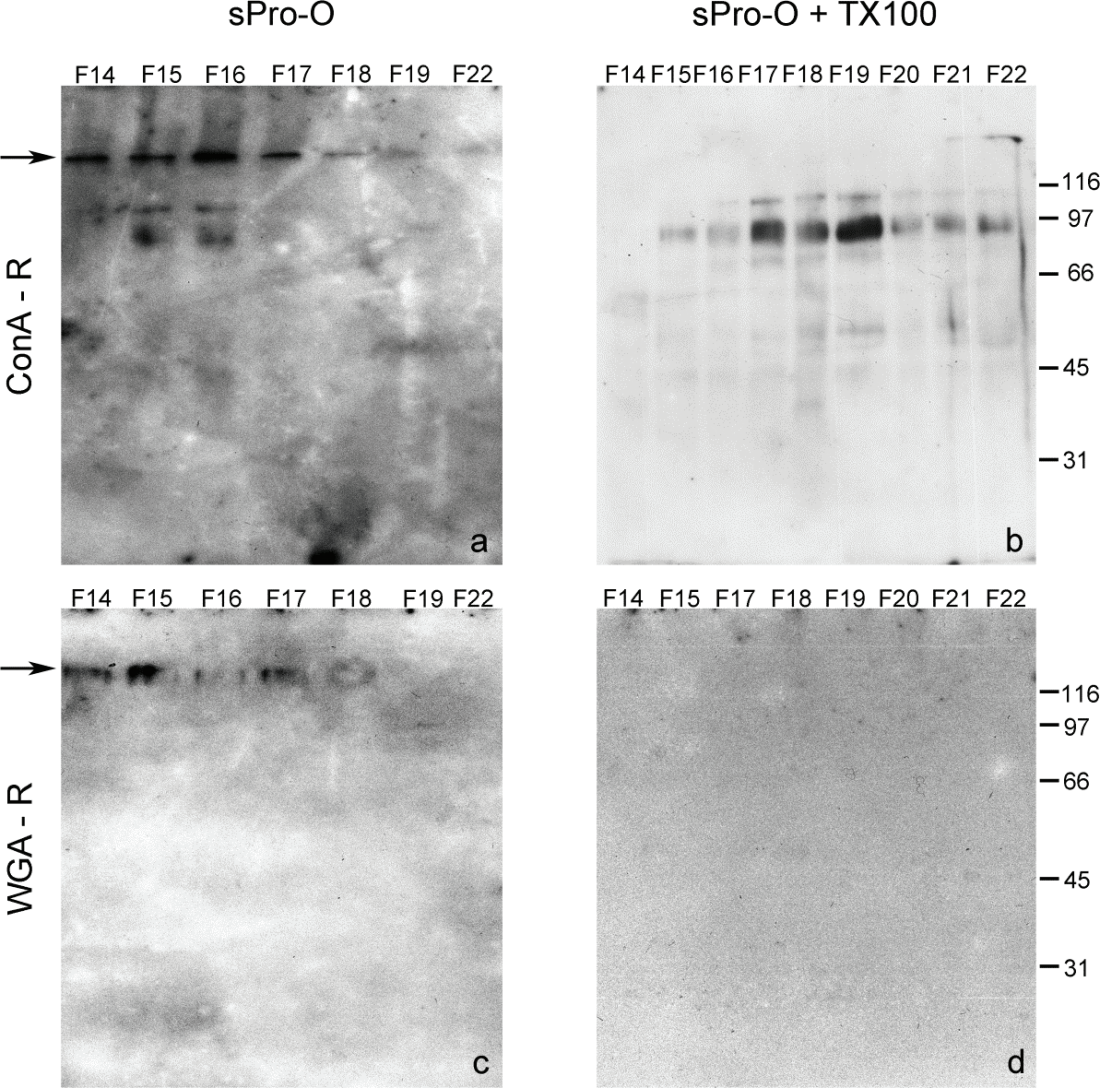
Distribution of Con A-reactive and WGA-reactive glycoproteins of the detergent-treated seminal prostasomes from oligozoospermic men. Selected gel filtration (Figure 5) fractions (F) of native prostasomes from human seminal plasma of oligozoospermic men (sPro-O) (a, c) and TX-100-treated sPro-O (b, d) were subjected to lectin blot using Con A (a, b) and WGA (c, d). Con A-R: concanavalin A-reactive glycoproteins; WGA-R: wheat germ agglutinin-reactive glycoproteins. The numbers (in kDa) indicate the position of molecular mass standards. Arrows indicate the border of stacking and separating gel.

In parallel, prostasomes isolated from human seminal plasma of oligozoospermic men (sPro-O) were subjected to TX-100 treatment and analyzed in the same manner. Compared to the native sample, the treated ones exhibited patterns of Con A- (Figure 5a) and WGA-reactive glycans (Figure 5b) as well as GGT (Figure 5c) that indicated decrease/loss and/or redistribution, similarly as found for sPro-N. In addition, similarity with sPro-N was also noticed regarding the effect on the redistribution of TS: CD63 and CD81 (Figure 5d). However, although CD9 (Figure 5d, fraction 14) and gal-3 (Figure 5d, fractions 14–16) both remained close to the initial position as observed for sPro-N, they exhibited partially overlapping profiles. Moreover, the immunoreactivity of both TS (CD63 and CD81) was barely detectable. Observation at the ultrastructural level suggested more general disruption of sPro-O (Figure 6) than sPro-N. In general, vesicular structures were low abundant and their morphology was clearly different from sPro-N. Integrity of the TX-100-treated vesicles reflected on patterns of total proteins (Figure 7) and glycoproteins (Figure 8) and indicated more severe perturbation than for sPro-N (Figure 3a). In comparison with the native sPro-O (Figure 7a), a significant loss of protein in the entire range of molecular masses, which remained mostly in the region below 66 kDa, was observed (Figure 7b). In agreement with this, Con A-reactive glycoproteins of native sPro-O (Figure 8a) were also strikingly decreased, including the major one at 97 kDa (Figure 8b). Moreover, lectin blot failed to detect any WGA-reactive glycoproteins in the TX-100 treated sPro-O (Figure 8b). Compared with those for sPro-N, the profiles of both types of released glycoproteins for sPro-O indicated more intensive aggregation to form complexes which, in general, interfere with or prevent the detection of contributing components.

## Discussion

The results obtained revealed novel patterns of surface-associated prostasomal proteins and related them to the molecular disposition on detergent-sensitive/resistant membrane domains that exist under normal physiology and conditions of low sperm count. In summary, distinct differences were found in the influence of detergent on solubilization of each tetraspanin as well as their relation with the other examined surface-associated molecules. Accordingly, they were grouped into two patterns mainly consisting of overlapped CD9/gal3/WGA-reactive glycoproteins and CD63/Con A-reactive glycoproteins/GGT. When the effect of TX-100 on sPro-N is compared with that on sPro-O, the overall similarity can be seen regarding the redistribution of examined surface-associated glycoproteins including GGT, all presumed to be mainly part of the vesicle coat. In contrast to this, greater difference was found in the redistribution of true integral membrane proteins exemplified by TS as well as gal-3, which could exist as membrane-associated through carbohydrate/protein-binding interactions. More specifically, in sPro-O, perturbation of CD9 and gal-3 was found to be related to engagement in different high molecular mass complexes and mutual segregation rather than co-localization in detergent-resistant membranous structures as could be deduced for sPro-N.

The existence of a TS-web on EVs, in general, has not been studied (4). In addition, there are few data on the composition of prostasomal surface glycans and gal-3 (2, 16). TS are a family of integral membrane proteins that may be involved in three levels of interaction with their molecular partners (11, 20, 21). As a result of these interactions, they are grouped into detergent-soluble tetraspanin-enriched membranes (1, 10, 22) that are clearly different from other types of higher order molecular complexes (23). Some detergents used for the investigation of prostasomal proteins (24) may differently affect TS interactions with their molecular partners. Since these interactions were out of the scope of this study, and we actually wanted to disrupt TS–TS interactions, we choose TX-100 treatment as the most commonly used method for the extraction of selected molecules (both TS and GGT) (15, 22). In addition to TS, the recruitment of different molecules into organized complexes may involve galectins (25, 26). Although soluble proteins, galectins, are readily found as membrane-associated through interactions achieved by carbohydrate-binding (cross-links N-glycans as ligands) or other protein- or lipid-binding domains, such as gal-3 as a distinct member of this family of lectins (5, 23, 27).

Thus, TS are expected to be readily solubilized, and since they are medium sized (~250 amino acids), this can cause a broad elution pattern due to the shift to higher molecular masses (depending on the composition of complexes with ligands). Indeed, the observed redistribution of CD63 was in agreement with this, suggesting abundant release in molecular complexes in response to detergent treatment of both sPro-N and sPro-O. However, one part of CD63-immunoreactivity remained at the initial position. In contrast to CD63, the elution of CD9 was only slightly shifted suggesting that it remains in high molecular mass complexes in both sPro-N and sPro-O. However, the complexes in sPro-N and sPro-O seemed to differ judging by the influence of detergent treatment on their structure. Thus, TEM suggested that CD9 in sPro-N remained in detergent-insoluble membranes/vesicular structures, whereas in sPro-O it was rather a part of aggregated protein complexes (both eluted in the void volume). This is supported by gal-3 distribution, which overlapped completely with CD9 in sPro-N but partially in sPro-O. The possibility of glycan-mediated or protein–protein interaction of gal-3 with CD9 can be supported by their detected co-localization, since neither type of interaction is expected to be influenced by TX-100. As for CD81, it could not be detected after TX-100 treatment in either sPro-N or sPro-O. This can be related to the data indicating that different antibodies differentially recognize CD81 if it is associated with the TS-web, or if the web is disrupted using the TX-100 (28). CD81 was previously reported to interact with GGT (29), which was also monitored. In relation to this, our results for the redistribution of prostasomal surface-associated GGT, monitored by measuring the enzymatic activity, clearly indicated its release from vesicles and appearance in molecular complexes (30). However, at this stage, it cannot be confirmed if this GGT pattern is due to its complex with CD81. Differences in the solubilizing properties of TS might be related to the facts that CD9 is a glycosylated proteolipid, CD63 is a glycosylated protein, and CD81 is a non-glycosylated protein (1). Thus, the TS structure itself and the specificities of the prostasomal surface microenvironment (in terms of distribution and presentation of glycans) may also influence the results obtained for detergent sensitivity. So far, it was reported that the examined TS and gal-3 are found co-isolated with prostasomal lipid rafts (9). It is known that lipid rafts contain an unusual lipid composition rich in cholesterol, which renders them insoluble upon detergent treatment (9, 31) and that they may associate with TS by lateral crosstalk between membrane domains. In relation to this, the existence of several prostasomal gal-3 isoelectric variants including a truncated form (carbohydrate recognition domain only) (7) which could reside in a different membrane microenvironment and consequently organize related but distinct molecular complexes may be responsible for specific redistribution profiles of glycoproteins/TS.

Detergent-soluble glycoproteins as molecules, which, on the one hand, can penetrate the membrane core and anchor hydrophobically, and, on the other hand, constitute a specific coat (32, 33), could also influence the stability and accessibility of the domain that may be intercalated with detergent. In this study, in intact sPro-N and sPro-O, WGA and Con A revealed a cluster of distinct partially overlapped glycoproteins. They were almost completely released from vesicles upon TX-100 treatment. However, the detergent-induced changes were distinct, influencing their detection depending on the experimental conditions used, especially for WGA-reactive ones. Significant shielding in auto-aggregates/heterologous complexes which can interfere with lectin binding, or mixed release of glycolipids/lipoproteins which escape detection by the methods used, is in agreement with the observed behavior, much emphasized in sPro-O. It is interesting that the majority of Con A-reactive glycoproteins were revealed in the region overlapping prostasome signature bands in both intact sPro-N and sPro-O. They are glycoproteins comprising the integral membrane protein CD13 of 150 kDa, transmembrane, and soluble CD26 of 82–110 kDa and soluble CD10 of 94 kDa (34). Regarding TS, all this indicated mixed patterns of different Con A-reactive glycoproteins and their preferential associations with CD63, which exhibited an overlapped solubilization pattern.

Analysis of membranous proteins is very difficult, since they usually exhibit anomalous behavior in many standardly used protein techniques (35–37). The possibility that some proteins could aggregate in spite of the critical micelle concentration, due to their abundance or inherent structure as well as that resolved peaks could be a set of peaks from protein, protein complexes, lipid and detergent, must be taken into consideration (38). Thus, the obtained molecular patterns themselves are descriptive and provide qualitative data. As a rule, they comprise numerous differently abundant bands. Some of them could have variable presence and some of them are constitutively present. Similar to the total prostasomal protein pattern exemplified by three prostasome signature bands (34), solubilization signature provided data in terms of annotation of main glycoproteins/TS to distinct detergent-sensitive or insensitive prostasomal patterns. Consequently, they can be used reliably for the comparison of prostasomes/any EVs (after establishing their own solubilization signature) in different physiological conditions. In terms of the presumed role of EVs as a communication tool (39, 40), these initial data could be a base for addressing the place of scaffolding in enabling the membrane functionality of EVs. In addition, it may initiate widening investigations on the basic issues of membrane complexity (41–43) usually deduced from the cell surface of plasma membrane to the field of EVs membrane.
